# Differences in the ability to predict and prepare for sexual activity between HIV-infected and HIV-uninfected young South African Women

**DOI:** 10.5897/JAHR2017.0455

**Published:** 2018-01-31

**Authors:** Rokhsanna Sadeghi, Amina Alio, Thola Bennie, Melissa Wallace, Shubing Cai, Beau Abar, Linda-Gail Bekker, David Adler

**Affiliations:** 1 Department of Emergency Medicine, University of Rochester, Rochester, NY, USA; 2 Department of Public Health Sciences, University of Rochester, Rochester, NY, USA; 3 Desmond Tutu HIV Centre, Institute of Infectious Diseases and Molecular Medicine, Faculty of Health Sciences, University of Cape Town, Cape Town, South Africa; 4 Desmond Tutu HIV Foundation, University of Cape Town, Cape Town, South Africa; 5 Department of Public Health Sciences, University of Rochester, Rochester, NY, USA

**Keywords:** Sexual behavior, HIV, young women, South Africa

## Abstract

The Human Research Council’s National HIV Prevalence, Incidence and Behavior Survey ranks South Africa first in HIV incidence in the world with 400,000 new infections in 2012 and found the HIV incidence rate among female youth aged 15 to 24 years to be 2.5% that year. The objective of this study was to compare the pattern and predictability of sexual activity between HIV-infected and HIV-uninfected young South African women. Sexually active young women between the ages of 16 and 21 years old completed a study survey between October 2012 and 2014 at two Desmond Tutu HIV Foundation centers. 100 young women with a mean age of 19.04 years responded to the survey. 51 women (51%) were HIV-infected and 49 were HIV-uninfected (49%). HIV-infected young women were found to be statistically less likely to have a temporal pattern to their sexual activity as compared to HIV-uninfected young women (56.9 vs. 95.9%, p<0.0001). While controlling for frequency of sex and lifetime sexual partners, HIV status remains a significant predictor of having a pattern of sexual activity (OR=16.13, p=0.0004) and a predictor of having sex on the weekend only (OR=4.41, p=0.0022). The ability to predict when sexual activity will occur enables a woman to prepare for its associated risks. HIV-uninfected young women are more likely to have a predictable pattern to their sexual activity as compared to HIV-infected young women. Knowledge of the sexual behavior patterns of this high-risk population will aid in the development of effective HIV prevention campaigns.

## INTRODUCTION

The Human Research Council’s National HIV Prevalence, Incidence and Behavior Survey ranks South Africa first in HIV incidence in the world with 400,000 new infections in 2012 (Shisana et al., 2014). Furthermore, according to UNAIDS (2013) estimates, there are 6,300,000 people living with HIV in South Africa with a 19.1% nationwide prevalence rate in adults aged 15 to 49 years old. At least half of all new HIV infections in the developing world were amongst youth and young adults (Gray, 2010). Understanding the temporal patterns of sexual activity and the ability of individuals to predict their sexual activity among this high-risk population will aid in the development of effective HIV prevention campaigns.

Current research highlights behaviors that put individuals at high risk of contracting HIV, but there is a paucity of studies that investigate and compare sexual behavior patterns between HIV-infected and HIV-uninfected young women. Increased risk of HIV transmission in young women has been found to be associated with greater than five lifetime sexual partners, having sex while under the influence of alcohol/drugs, less frequent condom use, prior pregnancy, and history of sexually transmitted infections (STIs) (Danielson et al., 2014). In addition, poor self-esteem and history of sexual or physical abuse has also been associated with higher risk of infection (Danielson et al., 2014). Young women may mistakenly perceive their risk of HIV transmission to be low given current monogamy, trust in current partner, and lack of injection drug use (Danielson et al., 2014; Overby and Kegeles, 1994). At risk young women have been found to be more concerned about issues relating to poverty, than HIV risk (Overby and Kegeles, 1994).

Knowledge of temporal patterns of sexual activity is highly relevant to HIV-prevention methods such as vaginal microbicides or female condoms. The ability to predict when sexual activity will occur enables a woman to prepare for its associated risks. In an environment of gender inequality, the success of female condom use in prevention of STIs has had limited success, largely due to lack of adherence and partner opposition (Bersinska et al., 2001). Trials to test for effectiveness of oral and vaginal anti-retroviral based pre-exposure prophylaxis methods for HIV prevention are on the rise (Obiero et al., 2012). These methods are thought to be easy to use and less apparent to partners, therefore, their use for transmission prevention has the potential to play a large role in curbing HIV infections in this high-risk population. One major factor in the success of HIV prevention methods is appropriate use and adherence by individuals at high risk for infection. Understanding sexual behaviorof high-risk young women will aid in developing targeted prevention strategies.

The objective of this study was to assess differences in the ability to predict the timing of sexual activity between HIV-infected and uninfected young women, in order to aid in planning a targeted approach to HIV prevention.

## METHODOLOGY

A cross-sectional survey study was conducted on sexually active, young women between the age of 16 and 21 years old from October 2012 through September 2014. No participants were married at the time of the study. Study participants were recruited from the Desmond Tutu HIV Foundation (DTHF) Youth Centre in Masiphumelele, South Africa and the Hannan-Crusaid Treatment Centre in Gugulethu, South Africa as part of a larger study assessing cervical dysplasia and high-risk human papilloma virus infections in this population. Cohort enrollment occurred sequentially until the goal of 100 participants was met. All participants in the parent study were included in the survey study. Survey information was collected through interviews conducted by community-based members of the study team.

All participants signed informed consent (aged 18 and older) or signed young assent documents (aged 16 and 17) to accompany parental consent forms in order to participate in this study. This study was approved by the Research Subjects Review Board at the University of Rochester and the Faculty of Health Sciences Human Research Ethics Committee at the University of Cape Town.

The survey included demographic information and factors relating to sexual behaviors, such as number of current, recent, and lifetime sexual partners, history of sexually transmitted infections, contraception methods, frequency of sex (average days per month), timing of sex, number of pregnancies, numbers of live birth, intention to have children, and who they ask regarding family planning. The primary outcome variable, timing of sex, was recorded as typical days of the week that participants engaged in sexual activity (Monday through Sunday), along with a response option for no particular pattern. These responses were further categorized into binomial groups regarding a discernable pattern to sexual activity (yes or no). Sexual activity on the weekend was defined Friday, Saturday, and/or Sunday. HIV status of study participants was confirmed upon study enrollment.

The data were analyzed through Chi-Squared statistics as well as multivariate analyses to control for potential confounders. SAS 9.3 (SAS Institute Inc, Cary, NC) was used to analyze data. Statistical significance level was placed at p ≤ 0.05.

## RESULTS

One hundred young women completed the survey. Table 1 describes the demographic and behavioral variables of the study participants. The mean age of participants was 19.04 years (range 16 to 21 years). Fifty-one (51%) women were HIV-infected and 49 (49%) women were HIV-uninfected.

Seventy-six (76%) study participants reported a discernable pattern to the timing of their sexual activity. Sixty-two (62%) participants reported having sexual activity on the weekends only. However, significant differences were identified between HIV-infected and HIV-uninfected participants. Forty-eight (96.0%) HIV-uninfected women reported a pattern to their sexual activity, whereas only 28 (56.0%) HIV-infected women reported a pattern to their sexual activity (p<0.001) (Table 2).

Thirty-nine (78.0%) HIV-uninfected women reported sexual activity only on the weekend, whereas only 23 (46.0%) of HIV-infected women reported sexual activity only on the weekend (p=0.001) (Table 2). When controlling the frequency of sex and number of lifetime sexual partners, HIV-uninfected status remained significantly associated with having a predictable pattern of sexual activity (OR 17.86, p<0.001) and predictor of sex on the weekend only (OR=4.06, p=0.003).

Our study participants reported using four main forms of contraception (Table 3). Although condom use was reported as the most commonly used form of contraception, the majority of participants, 54 (54%), reported inconsistent condom use (Table 3). Consistency of condom use varied significantly between HIV-uninfected and HIV-infected women, 15 (30.0%) HIV-uninfected women reported consistent condom use and 31 (62.0%) HIV-infected women report consistent condom use (p=0.001). Among the HIV-uninfected participants, 14 (29.8%) women who reported a temporal pattern to sexual activity also reported consistent condom use, whereas none (0%) of the women without a pattern reported consistent condom use (p=0.345). Among the HIV-infected women, there was no statistically significant association between the consistent use of condoms and having a temporal pattern to sexual activity. Sixteen (57.1%) HIV-infected women who reported a pattern also reported consistent condom use, whereas 15 (68.2%).

HIV-infected women without a pattern reported consistent condom use (p= 0.425).

Significant differences were identified between having a temporal pattern to sexual activity and reported history of STIs. Sixteen (66.7%) women with no pattern to their sexual activity reported a history of STI, whereas 31 (40.8%) women with a pattern to their sexual activity reported a history of STI (p=0.027). Fourteen (28.6%) HIV-uninfected women reported a history of STI as compared to 33 (64.7%) HIV-infected women (p<0.001).

The probable source of transmission of HIV in participants was through behavioral mechanisms in 41 participants and perinatally in 10 participants. Analyses excluding the perinatally infected women did not affect statistical significance in the pattern of sexual activity and sexual activity on weekends only between the HIV-infected women and the HIV-uninfected women. In this sub-analysis, HIV-uninfected women continue to have more consistent patterns to their sexual activity, even while controlling for frequency of sexual activity and number of partners.

## DISCUSSION

In South Africa, there is an HIV epidemic that has been particularly severe among young people (Gray, 2010). There are several sociocultural barriers that prohibit available preventative methods from being used consistently or effectively, such as gender inequality, attitudes toward prevention methods, poverty, lack of knowledge, and limited access to resources (Danielson et al., 2014; Overby and Kegeles, 1994; Dunkle et al., 2004). These past studies suggests that some women have poor insight into potential transmission risks and may engage in high-risk behavior while on alcohol/drugs, which may be unplanned and at arbitrary times.

Gender inequality and gender-based violence in South Africa has also been recognized to be an important determinant of women’s HIV risk (Boer and Mashamba, 2005; Pettifor et al., 2004; Wood et al., 1998). It has been found that women with violent and controlling male partners are at increased risk of HIV infection. This is thought to be secondary to higher HIV rates in abusive men, as well as an increased likelihood for these men to impose high-risk practices on their partners (Dunkle et al., 2004). Innovative public health campaigns for the reduction of HIV transmission are needed to implement strategies that will specifically target high-risk groups.

This study included young women recruited from townships outside of Cape Town, South Africa where the overall prevalence of HIV is 23% (The Desmond Tutu HIV Foundation, Youth Centre, 2015b; The Desmond Tutu HIV Foundation, Hannan-Cruisad Treatment Centre, 2015a). This cohort of young women represents a high-risk population for HIV transmission. A statistically significant difference was found in the pattern of sexual activity and sexual activity on weekends only between HIV-infected women and HIV-uninfected women. HIV-uninfected women were found to have more consistently predictable patterns to their sexual activity, even while controlling for frequency of sexual activity and number of partners. HIV-uninfected women were found to predominantly engage in sexual activity on the weekends. Although prior research has found that adolescent women with sexually transmitted infections are most likely to have sexual intercourse on Fridays and Saturdays relative to other days of the week, no study has been found that directly compares these differences between HIV-infected and HIV-uninfected women (Fortenberry, 1997).

The results of this study also demonstrate that HIV-uninfected women who have a pattern to their sexual activity are statistically more likely to consistently use condoms as compared to HIV-uninfected women who do not have a pattern to their sexual activity. Although, the overall rate of consistent condom use was found to be low for HIV-infected and HIV-uninfected women (62.8 and 28.6%, respectively). This is consistent with existing literature that reports 43% of HIV-infected adolescents and 77% of South African youth use condoms inconsistently (Pettifor et al., 2005;Murphy, et al., 2001).

It was also found that predictability of sexual activity is correlated with a history of STI. Nearly 67% of young women without a pattern to their sexual activity report a history of STI, whereas only 41% women with a pattern to their sexual activity report a history of STI. These findings likely relate to similar behavioral characteristics that place young women at risk for acquisition of HIV.

The findings of this study demonstrate that there is greater predictability of timing of sexual activity among young HIV-uninfected women as compared to their HIV-uninfected counterparts. This predictability may allow young women to be better prepared to prevent HIV transmission and account, in part, for their HIV-uninfected status. At the public health intervention level, understanding young women’s ability to predict when they have sex could enhance prevention strategies that depend on preparation for sexual encounters.

The ability to predict sexual behavior patterns is crucial for the design of studies assessing the feasibility, acceptability and effectiveness of pre-exposure prophylaxis methods currently being conducted with young women. This information will inform study development for safety and efficacy of various dosing schedules of oral and vaginal pre-exposure prophylaxis products and oral and vaginal microbicides. These methods are likely to become a mainstay of prevention of HIV transmission as their use is controlled by women (Mutua et al., 2012; Elias and Coggins, 1996).

Knowledge regarding HIV transmission alone does not change the behavior of young women; therefore, an effective public health prevention program should be based on models and theories of behavior in order for programs to be designed to change those factors that lead to the undesirable risky behaviors (Boyer and Kegeles, 1991). Given that the vast majority (95.9%) of HIV-uninfected young women have a pattern to their sexual activity, this high-risk population would especially benefit from a targeted HIV transmission prevention method.

The strength of this study is the ability to analyze behavioral data in a particularly vulnerable population. This data will add to a growing body of patient-centered studies, which ultimately aim at reducing morbidity and mortality of young women at risk for HIV. The predictability of the timing of sexual activity in HIV-infected and HIV-uninfected young women is yet to be studied. Nonetheless, there are important limitations to this study. First, the sample size limits the ability to power sub-analyses within the cohort including a comparison between the participants recruited from the DTHF Youth Centre and the Hannan-Crusaid Treatment Centre. Despite this limitation, these two study sites are demographically similar, ethnically homogeneous (Xhosa), and are located within close proximity to each other, and have the same socioeconomic profile. Therefore, there are unlikely to be major behavioral differences between women recruited from these two centers. Another limitation is the potentially reduced reliability of self-reported responses from young women regarding sexual behaviors. To limit this, interviews were conducted in a confidential manner.

Further studies should be conducted in order to gain additional insight to sexual behavior patterns. A mixed-methods approach may accomplish this by obtaining a thorough understanding within the context of the theory of planned behavior by investigating the interactions between behavior, environment, and the individual, as well as perceived power and perceptions of related external factors and objective realities (DiClement et al., 2013).

## Conclusion

The ability to predict when sexual activity will occur enables a woman to prepare for its associated risks. HIV-uninfected young women are more likely to have a predictable pattern to their sexual activity as compared to HIV-infected young women. Findings from this study can help to anticipate the sexual behaviors of women and inform interventions to ensure that young women are prepared to use effective HIV prevention methods when the need arises. This information can be utilized in the development of theory based prevention programs.

## Figures and Tables

**Table 1:** Demographic and behavioral variables of study participants.

Variable	HIV-infected (n = 50)		HIV-uninfected (n = 50)		p-value

	Frequency (%)	M (SD)	Frequency (%)	M (SD)	
Age in years	-	19.64 (1.40)	-	18.44 (1.40)	<0.001
Frequency of sex (days per month)	-	6.12 (4.75)	-	5.68 (4.41)	0.632
Ever used tobacco^a^	6 (12)	-	3 (6)	-	0.280
Number of sexual partners - lifetime	1 = 11 (22)		1 = 4 (8)	-	0.054
	2–5 = 34 (68)		2–5 = 44 (88)		
	>5 = 5 (10)		>5 = 2 (4)		
Number of sexual partners – past 6 months	1 = 48 (96)		1 = 47 (94)	-	0.646
	2–5 = 2 (4)		2–5 = 3 (6)		
History of STI	33 (66)		14 (28)	-	<0.001

a Percentages are based on nonmissing data.

**Table 2:** Young women with a pattern to sexual activity and engaging in sexual activity only on the weekend.

Variable	HIV-infected, n = 51, Frequency (%)	HIV-uninfected, n = 49, Frequency (%)	p value
Pattern to sexual activity	28 (56.0)	48 (96.0)	<0.001
Sexual activity only on the weekend	39 (78.0)	23 (46.0)	0.001

**Table 3:** Current contraceptive method used and frequency of condom use.

Current contraception^a^	Frequency (%)		p value
	HIV-infected	HIV-uninfected	
Condom	49 (98.0)	41 (82.0)	0.008
Injection	29 (58.0)	32 (64.0)	0.539
Pill	1 (2.0)	3 (6.0)	0.307
Subdermal implant	0 (0.0)	1 (2.0)	0.315
None	1 (2.0)	1 (2.0)	1.00
**Frequency of condom use**			
Always	31 (62.0)	15 (30.0)	0.001
Most of time	8 (16.0)	18 (36.0)	0.023
Hardly ever	10 (20.0)	15 (30.0)	0.248
Never	1 (2.0)	2 (4.0)	0.557

a Frequency exceeds 100 because many participants use more than one contraceptive method.
